# *ENPP2* Methylation in Health and Cancer

**DOI:** 10.3390/ijms222111958

**Published:** 2021-11-04

**Authors:** Maria Panagopoulou, Dionysios Fanidis, Vassilis Aidinis, Ekaterini Chatzaki

**Affiliations:** 1 Laboratory of Pharmacology, Medical School, Democritus University of Thrace, GR-68100 Alexandroupolis, Greece; mpanagop@med.duth.gr; 2 Biomedical Sciences Research Center Alexander Fleming, Institute of Bio-Innovation, GR-16672 Athens, Greece; fanidis@fleming.gr (D.F.); aidinis@fleming.gr (V.A.); 3 Institute of Agri-Food and Life Sciences, Hellenic Mediterranean University Research Centre, GR-71410 Heraklion, Greece

**Keywords:** autotaxin, *ENPP2*, methylation, cancer, expression, regulation

## Abstract

Autotaxin (ATX) encoded by Ectonucleotide Pyrophosphatase/Phosphodiesterase 2 (*ENPP2*) is a key enzyme in Lysophosphatidic Acid (LPA) synthesis implicated in cancer. Although its aberrant expression has been reported, *ENPP2* methylation profiles in health and malignancy are not described. We examined in silico the methylation of *ENPP2* analyzing publicly available methylome datasets, to identify Differentially Methylated CpGs (DMCs) which were then correlated with expression at gene and isoform levels. Significance indication was set to be FDR corrected *p*-value < 0.05. Healthy tissues presented methylation in all gene body CGs and lower levels in Promoter Associated (PA) regions, whereas in the majority of the tumors examined (HCC, melanoma, CRC, LC and PC) the methylation pattern was reversed. DMCs identified in the promoter were located in sites recognized by multiple transcription factors, suggesting involvement in gene expression. Alterations in methylation were correlated to an aggressive phenotype in cancer cell lines. In prostate and lung adenocarcinomas, increased methylation of PA CGs was correlated to decreased *ENPP2* mRNA expression and to poor prognosis parameters. Collectively, our results corroborate that methylation is an active level of ATX expression regulation in cancer. Our study provides an extended description of the methylation status of *ENPP2* in health and cancer and points out specific DMCs of value as prognostic biomarkers.

## 1. Introduction

ATX encoded by *ENPP2* is a secreted lysophospholipase D (lysoPLD) and belongs to the ENPP (1–7) protein family [1]. ATX is responsible for the synthesis of the majority of extracellular LPA in blood. LPA acts locally upon increased ATX levels through at least six G protein-coupled receptors [2]. Increased ATX activity and levels have been correlated with several inflammatory [3] and fibroproliferative conditions [4], as well as with cancer [5]. In particular, increased expression of ATX in blood and the subsequent increase of LPA have been correlated with cancer invasiveness [6]. In addition, it has been shown that ATX expression is upregulated in cancerous [7,8] and fibrotic tissues [9].

*ENPP2* contains 26 introns and 27 exons and is located in the human chromosomal region 8q24 [10], a region with frequent genetic alterations in many cancers [11]. *ENPP2* is characterized by alternative splicing of mRNA. The best-known splice variants of *ENPP2* are isoforms alpha, beta and gamma; between them, differences in the stability and expression pattern have been documented among several tissues [12].

Epigenetic regulation of *ENPP2* has been previously reported [13]. DNA methylation, a well-studied epigenetic mechanism, can regulate gene expression [14], and aberrant gene-specific methylation has been correlated with many pathologies, such as cancer [15,16,17,18,19,20]. However, data on the methylation profile of *ENPP2* in health and pathology are fragmented. A study in Biliary Atresia (BA) showed hypomethylation at four CpGs of *ENPP2* promoter in the blood and liver of BA patients in relation to normal tissue and was correlated to increased ATX expression [21]. *ENPP2* promoter hypermethylation and gene under-expression was found in primary invasive breast carcinomas [13]. Similarly, in breast cancer cell lines, a promoter-associated CpG (cg02156680) of *ENPP2* was found highly methylated [22].

**Figure 1:**
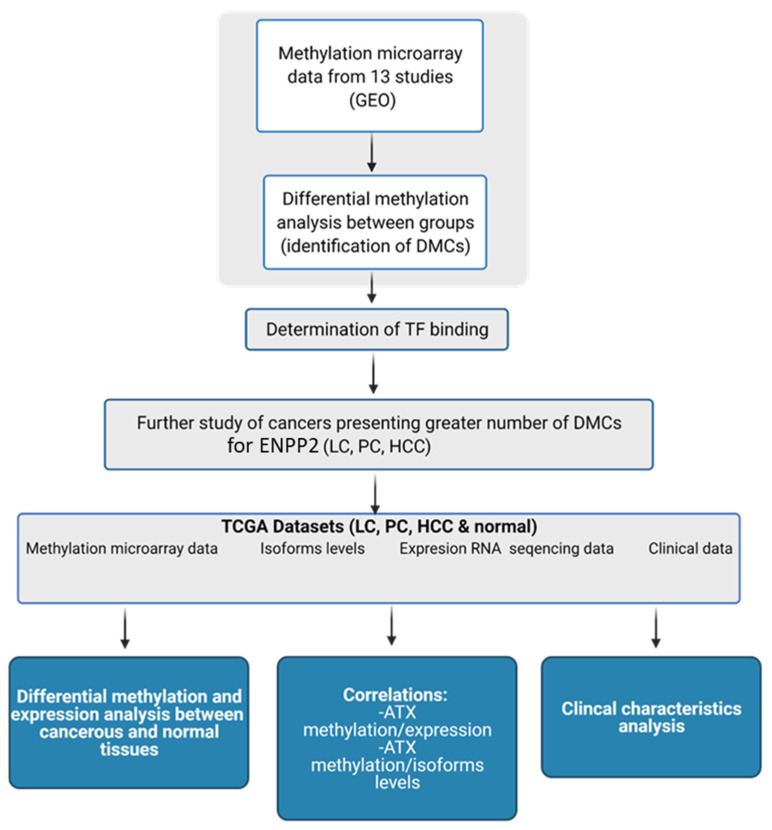
Workflow of the study of *ENPP2*. Created with BioRender.com (Agreement number: UW22ZTY5U7) (accessed on 24 September 2021). Abbreviations: GEO: Gene Expression Omnibus, DMCs: Differential Methylated CpG sites, TF: Transcription Factor, TCGA: The Cancer Genome Atlas, LC: Lung Cancer, PC: Prostate Cancer, HCC: Hepatocellular Carcinoma, ATX: Autotaxin.

In the present study, we studied in silico the methylation of *ENPP2* in health and several malignancies and correlated it with gene and isoform expression, aggressiveness and prognosis. Cancer types included in our study were chosen based on their high world incidence, mortality and prevalence [23], as well as access to readily available suitable high-throughput datasets. We examined publicly available methylation datasets from readings by the Illumina methylation bead-chip arrays found in *Gene Expression Omnibus* (GEO), to identify Differentially Methylated CpGs (DMCs) of *ENPP2* between health and disease. Lung, prostate and liver cancer presented a greater number of Promoter Associated (PA) DMCs for *ENPP2* and were further pursued using large datasets retrieved from The Cancer Genome Atlas (TCGA), which allowed DMC correlation to clinicopathological parameters and gene expression. A workflow of our study is presented in Figure 1.

## 2. Materials and Methods

### 2.1. GEO Datasets for the In-Silico Methylation Analysis

DNA methylation data from cancer cell lines and patients with different malignancies and corresponding controls were obtained from the GEO (Gene Expression Omnibus) (https://www.ncbi.nlm.nih.gov/geo/, accessed on 25 December 2020) database [24]. Colorectal Cancer (CRC), Lung Cancer (LC), melanoma, Prostate Cancer (PC), Gastric Cancer (GC), Liver Cancer (HCC), cell lines and normal tissues were used as keywords in GEO query and ‘Methylation profiling by array’ as study type. A total of 73 studies were found; between them, only those using Infinium Human Methylation 27 K, 450 K and EPIC BeadChips (San Diego, CA, USA) and providing adequate data were selected for further analysis. In total, 13 studies, GSE27130 [25], GSE98534 [26], GSE63704, GSE46306 [27], GSE134772 [28], GSE120878 [29], GSE76938 [30], GSE97686 [31], GSE113017 [32], GSE113019 [32], GSE71627 [33], GSE50192 [34,35] and GSE51815 [36], were recruited for our analysis. Descriptions of study groups and correlations are presented in Table 1.

### 2.2. Methylation and Statistical Analysis

Methylation analysis was carried out using normalized beta values ranging between 0 (no methylation) and 1 (full methylation) representing methylation levels of each CpG site (Level 3 data). The Kolmogorov–Smirnov test was applied to check for normality in distribution. Statistical analysis was performed using IBM SPSS 19.0 statistical software (IBM Corp. 2010. IBM SPSS Statistics for Windows, Version 19.0. Armonk, NY, USA). One-Way ANOVA tests followed by Bonferroni post-hoc or Kruskal–Wallis tests were used for comparisons of continuous variables among three or more different groups. In the case of binary variables, *t*-test or Mann–Whitney tests were also applied. Pearson or Spearman correlation was applied to compare two continuous variables. Differentially Methylated CpGs (DMCs) for *ENPP2* were identified based on the False Discovery Rate (FDR—adjusted *p*-value < 5.00 × 10^−2^).

### 2.3. In Silico Determination of Transcription Factor (TF) Binding

In order to examine if the DMCs identified were correlated to *ENPP2* expression, we further analyzed promoter regions to locate TF binding sites. Hence, PROMO (http://alggen.lsi.upc.es/, accessed on 18 March 2021) [37] tool was used in order to define possible TFs binding in identified DMCs inside *ENPP2* promoter. Only human factors and human sites were considered for a TFs search.

### 2.4. Expression and Methylation Correlation Analysis Using TCGA Datasets

Normalized (gene and isoform level) RNA-seq (Illumina HiSeq), level 3 methylation legacy data (Infinium Human Methylation 450 K bead-chip) and corresponding available clinical data were retrieved from prostate adenocarcinoma, lung adenocarcinoma and liver hepatocellular carcinoma TCGA projects representing PC, LC and HCC cases, using the TCGAbiolinks R package [38]. In total, 212 LC, 235 PC and 241 HCC cancer cases along with adjacent healthy lung (15 cases), prostate (35 cases) and liver (42 cases) tissues were obtained. More specifically, cases were chosen to include both mRNA expression (gene and isoforms) and methylation data, from which all matched control and tumor cases were retrieved along with 200 additional tumor samples per cancer type. In the rare case of a case ID being represented by more than one methylation or expression file, the weighted average of the respective values was used for downstream analysis (all weights sum up to the unit). Spearman correlation was calculated per cancer type using the cor.test function. The cutoff level of significance was set to be FDR corrected *p*-value < 0.05. Last, *p*-values of linear models fitted between methylation and expression levels (*lm* R function) were used to test and establish the importance of small correlation coefficients. Analyses were performed using R version 4.0.4.

### 2.5. Expression, Methylation and Survival Analysis Using the UALCAN Database

In order to further verify our results, we used the UALCAN database (http://ualcan.path.uab.edu/, accessed on 10 September 2021) [39] that enables researchers to analyze cancer archived omics data. We performed expression, methylation and survival analysis of *ENPP2* in the three types of cancer used in our analysis (PC, LC, HCC) along with the corresponding controls. According to UALCAN, different beta value cut-offs have been considered to indicate hyper-methylation [beta value: 0.7 − 0.5] or hypo-methylation [beta-value: 0.3 − 0.25]. For mRNA expression, methylation and survival, we used TCGA gene analysis, and the screening conditions were as follows: gene “*ENPP2*”, TCGA dataset “Prostate adenocarcinoma”, “Lung adenocarcinoma”, “Lung Squamous cell carcinoma”, “Liver hepatocellular carcinoma”. We then used “expression”, “methylation” and “survival” as links for analysis of each cancer. Protein expression data were available only for lung adenocarcinoma, and Clinical Proteomic Tumor Analysis Consortium (CPTAC) datasets were used for analysis. For protein analysis, Z-values represent standard deviations from the median across samples for the given cancer type. Log2 Spectral count ratio values from CPTAC were first normalized within each sample profile and then normalized across samples.

## 3. Results

### 3.1. Analysis of ENPP2 Methylation from GEO Datasets

In silico methylation analysis of *ENPP2* was performed using methylome data retrieved using the GEO. The results are described below.

### 3.2. ENPP2 Methylation in Normal Tissues

**Figure 2:**
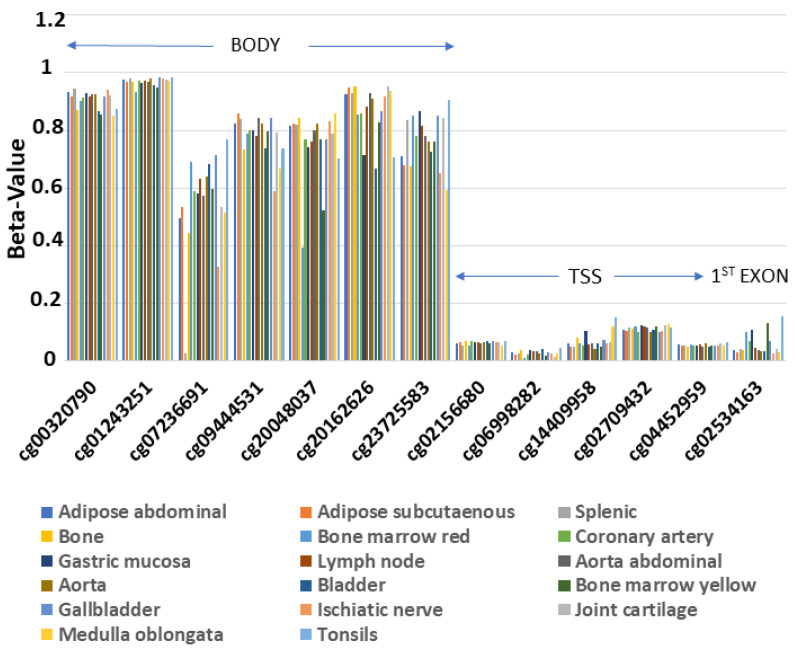
Similar methylation pattern of ENPP2 across 17 healthy tissues. Gene body region presents higher methylation levels in relation to TSS or 1st exon. Abbreviations: TSS = Transcription Start Site.

In order to examine the methylation profile of *ENPP2* across different human healthy tissues, we analyzed methylome data from 17 healthy tissues included in the GSE50192 study. We observed a consistent methylation pattern across all studied tissues (Figure 2), with methylation being increased in all 7 CGs in the gene body region and decreased in 5 CGs in the Transcription Start Site (TSS) and 1 in the 1st exon.

### 3.3. ENPP2 Methylation in Tissues from Cancer Patients

In order to unravel aberrant *ENPP2* methylation in cancer, we compared methylomes of malignant vs. respective benign tissues from 7 different cancer types, using 10 GEO datasets (GSE113017, GSE113019, GSE120878, GSE27130, GSE63704, GSE76938, GSE98534, GSE46306, GSE134772, GSE97686) (Table 1). In total, 13 DMCs were identified in 5 cancers, i.e., HCC (12 DMCs), PC (10 DMCs), LC (9 DMCs), melanoma (7 DMCs), CRC (1 DMC) (Table 2), most of which were common between them (Table 3), whereas no DMCs were identified in Precancerous Interepithelial Cervical Neoplasia (CIN) and Cancer (CC) and in Gastric Cancer (GC). With two exceptions, all gene body DMCs showed decreased methylation in cancer in relation to their controls. Most importantly, all CGs located in the promoter-associated region and the 1st exon, regions known to hold an important role in transcriptional regulation [38,40] were DMCs across different cancer types, all presenting increased methylation. These results demonstrate aberrant methylation of *ENPP2* in the majority of cancer types studied, following a specific pattern pointing to down-regulation of expression.

### 3.4. ENPP2 Methylation Was Correlated to Aggressiveness in Cancer Cell Lines

In order to study any relation of *ENPP2* methylation to cancer aggressiveness, we compared cell lines from hepatocellular and prostate cancer presenting a more (SKHEP1 and PC3 respectively) or less (HEPG2 and LNCAP respectively) invasive phenotype, using the GSE71627 study dataset [33]. In total, 12 DMCs were identified (Table 4), 6 of which were common in both cancer types. All 8 DMCs identified in HCC cell lines were also found in the liver tumor methylomes, whereas the common DMCs between prostate tissues and cell lines were 6/8. With two exceptions, all DMCs across the whole gene (1st exon, TSS, body) showed higher methylation in the more aggressive hepatocellular and prostate cell lines. These observations suggest an involvement of *ENPP2* methylation in cancer aggressiveness.

Interestingly, treatment of colon cancer cell lines with the DNA methylation inhibitor 5-aza-2′-deoxycytidine (GSE51815 study) caused a decrease of methylation in all 14 DMCs located throughout *ENPP2* (Supplementary Table S1), implying that methylation could present a potential therapeutic target to reverse the aggressive phenotype.

### 3.5. In Silico Analysis of TF Binding on the ENPP2 Promoter

**Figure 3:**
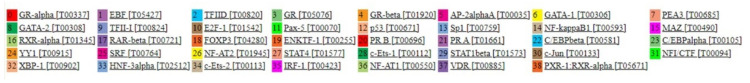
39 Transcription factors that can bind to the ENPP2 promoter in sites containing DMSs identified in cancer, with a dissimilarity margin ≤ 15%.

Regulation of gene expression via DNA methylation occurs mainly by disturbing TF and RNA polymerase binding to sites known to be necessary for initiation of transcription [41]. To support that the identified DMCs on *ENPP2* may actually play a role in regulating ATX expression, we examined if they are located within TF binding promoter regions that could initiate transcription. Analysis using the PROMO tool predicted 39 putative TFs that could bind to the *ENPP2* promoter (Figure 3), 4 of them (TFIID, GR, GR-beta, C/EBPbeta) on or in proximity to cg04452959, 7 TFs (TFII-I, GR-alpha, GATA-1, E2F-1, Pax-5, p53, Sp1) in cg02709432, 7 TFs (C/EBPbeta, C/EBPalpha, Pax-5, p53, ENKTF-1, YY1, GR-beta) in cg02156680 and 3 TFs (PEA3, GATA-1, XBP-1) in cg06998282. Interestingly, those 4 CGs located 200 nucleotides upstream of and up to the TSS (TSS200) (first 2), or 200 to 1500 nucleotides upstream of the TSS (TSS1500) (last 2) were identified as DMCs in most of the malignancies examined and between more and less aggressive cell lines. Collectively, these findings show that DMCs identified in the *ENPP2* promoter in cancer are found in sites significant for TF binding, and therefore, altered methylation is likely to affect transcription and expression of ATX.

### 3.6. ENPP2 Methylation and Expression Analysis from TCGA Datasets

An important objective of our study was to address if and how aberrant methylation of *ENPP2* is related to alterations in gene expression. Based on our findings, three cancer entities presenting the highest number of DMCs were selected for further study, in order to confirm altered *ENPP2* methylation in larger cohorts and correlate them with expression at gene and isoform levels. For this purpose, several available datasets including PC, LC and HCC readings of mRNA expression (gene and isoforms) and methylation along with the available clinical and demographic data were downloaded from TCGA.

### 3.7. ENPP2 Methylation and Expression Analysis in Prostate Cancer

Comparisons were performed between 235 prostate adenocarcinoma tumors and 35 healthy prostate tissues (Table 5). In general, results confirmed those from the GEO datasets. In total, 12 DMCs were identified between cancer and control tissues (5 in TSS, 1 in 1st exon and 6 in the gene body), 10 of which were common to those found in the GSE76938 dataset. All DMCs in TSS and the 1st exon presented increased methylation in PC in relation to controls, whereas decreased methylation was noticed in 3 out of 6 DMCs in the gene body area. Following this, we examined correlation of DMCs to clinicopathological patient characteristics, to reveal associations with prognosis. Methylation analysis in relation to available patient data (age, race, nodal status, relapse, tumor size and treatment response) showed a significant correlation with tumor size, as increased methylation of 3 CGs, namely, cg02534163 (1st exon), cg02709432 (TSS200) and cg23725583 (gene body), was found in larger tumors in relation to smaller tumors (*p* = 0.032). Furthermore, non-response to pharmacotherapy was correlated with increased methylation of cg01243251 in the gene body region (*p* = 0.023). No other correlations were found in relation to age, race, nodal status and the event of relapse.

**Figure 4:**
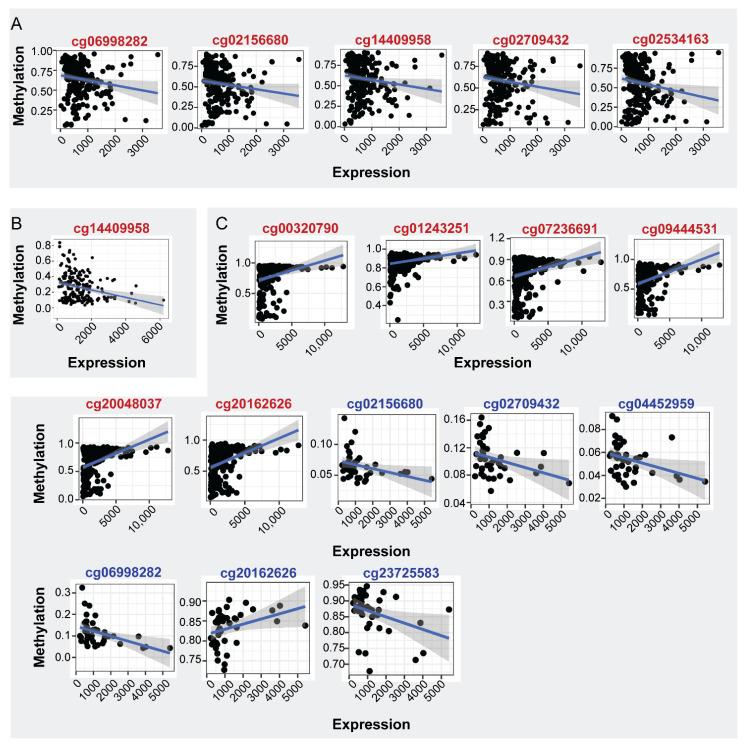
Correlation of ENPP2 CGs methylation and mRNA expression. CGs showing significant correlations are depicted (*p* < 0.05). (**A**) Expression-methylation scatter plots of CG sites of PC and normal samples, (**B**) expression-methylation scatter plots of CG sites of LC and normal samples, (**C**) expression-methylation scatter plots of CG sites of HCC and normal samples. Correlations from cancer and healthy samples are marked in red and blue CG color font, respectively.

mRNA expression analysis in the same samples showed decreased levels in PC in relation to normal tissues (LogFC: −0.379, FDR: 3.70 × 10^−2^), indicating that the increased methylation of *ENPP2* in PA regions is correlated with the decreased expression of *ENPP2* in PC. Spearman correlation of mRNA ENPP2 expression (at gene and isoform level) per CG site revealed statistically significant correlations shown in Figure 4A and Table 6. Between gene body CGs, a tendency towards positive correlation of mRNA expression to cg01243251 and cg20162626 methylation was observed and a negative to cg07236691. TSS CG sites cg02156680, cg02709432, cg06998282, cg14409958 and 1st exon cg02534163 methylation showed a negative correlation with expression, showing that the increased methylation at these regions is associated with decreased expression. Interestingly, although Spearman’s coefficient is relatively small for TSS and 1st exon CGs, successful fit of a linear model further supports the existence of an expression-methylation relationship (Table 6, coefficient *p*-value column). No significant correlations emerged between *ENPP2* expression and methylation in control prostate tissue.

In order to unfold the impact of CG methylation on *ENPP2* isoform expression [12,42], we downloaded mRNA expression data from *ENPP2* isoforms, i.e., isoform alpha (uc003yos.1), isoform beta (uc003yor.1 and uc003yot.1) and isoform gamma (uc010mdd.1). Uc003yot.1, uc003yos.1 and uc003yor.1 isoform expression showed statistically significant correlation with the methylation of several CGs. In specific, although they were all characterized by small effect sizes, uc003yor.1 expression is linearly related to the methylation levels of TSS and 1st exon CGs cg02156680, cg06998282, cg14409958 and cg02534163, respectively, further strengthening the observed correlation (Supplementary Figure S1 and Table S2). Finally, no relation emerged between expression of any of the *ENPP2* isoforms and methylation of CGs in healthy prostate samples, consistent with the observations at the gene level.

### 3.8. ENPP2 Methylation and Expression Analysis in Lung Cancer

Analysis was performed between 212 LC adenocarcinoma tumors and 15 healthy lung tissues, and results presenting statistically significant correlations are shown in Table 7. Findings confirmed those from the GEO datasets. Eight DMCs were identified between cancer and control tissues (3 in the TSS, 1 in the 1st exon, 4 in the gene body) and 6 of them were common to those found in the GSE76938 dataset. DMCs of *ENPP2* showed upregulation of methylation in TSS (cg04452959, cg06998282, cg14409958) and the 1st exon (cg02534163) and downregulation in the gene body (cg07236691, cg09444531, cg20048037, cg20162626). Methylation was also correlated to the available clinicopathological characteristics of LC and normal lung tissue samples (gender, age, nodal status, distance metastasis, relapse, tumor size and treatment response and stage). In LC samples, increased methylation of cg14409958 (TSS) was significantly correlated with advanced cancer stage (*p* = 0.035).

Differential mRNA expression analysis in the same samples showed decreased levels in LC in relation to normal tissues (LogFC: 1.285, FDR: < 1.00 × 10^−2^) similarly to PC, indicating that in cancer the increased methylation of PA CGs is correlated to decreased autotaxin expression. The impact of *ENPP2* methylation on its expression was examined in LC and healthy lung tissue samples. Spearman correlation of mRNA expression per CG resulted in a single statistically significant correlation (Figure 4B and Table 6). A reverse correlation was noticed between methylation of cg14409958 (TSS) and mRNA expression, suggesting again the DNA methylation role in repressing expression. Fit of a linear model once again reinforced the observed correlation. On the other hand, control samples did not show any statistically significant correlation after *p*-value correction, and only the methylation of body site cg7236691 showed a significant correlation coefficient along with a linear relationship to *ENPP2* expression levels. Last, no significant correlations were witnessed between methylation and expression levels of all isoforms examined, yet large rho values and significant linear model fit propose the existence of such a relationship.

### 3.9. ENPP2 Methylation and Expression Analysis in Hepatocellular Carcinoma

Analysis was performed between 241 HCC tumors and 42 control liver tissues. Statistically significant correlations are presented in Table 8. In total, 13 DMCs were identified between cancer and control (5 in the TSS, 1 in the 1st exon, 7 in the gene body) and 12 were common to those found in GSE113017 and GSE113019. Again, downregulation of methylation was noticed in all gene body CGs (cg00320790, cg23725583 and cg01243251) and upregulation of methylation in all TSS and 1st exon related CGs (cg02156680, cg02534163, cg02709432, cg04452959, cg06998282 and cg14409958) in HCC.

Methylation of *ENPP2* was also correlated to available clinical and demographic characteristics of the HCC cohort. Interestingly, in the tumor samples, increased methylation of the majority of the *ENPP2* CGs (cg00320790, cg01243251, cg02156680, cg02709432, cg07236691, cg09444531, cg14409958, cg20048037, cg20162626, cg23725583) (all *p* < 0.05) was noticed in women in relation to men. In addition, a negative correlation was found between age and methylation of cg00320790, cg01243251, cg07236691, cg09444531, cg20048037, cg20162626 and cg23725583 (all *p* < 0.001), i.e., younger people presented increased methylation in relation to older. Finally, increased methylation of cg04452959 was correlated to tumors with macro invasion in relation to those with no or micro invasion (*p* = 0.044). No correlation was noticed between methylation and BMI, hepatic inflammation, Ishak fibrosis, relapse, family history, grade, stage or tumor size. Analysis in normal samples showed a gender correlation only for one CG (cg20048037) which presented increased methylation in females (*p* = 0.033) in relation to males. Negative correlation was also noticed between cg01243251 methylation and age (*p* = 0.037). Finally, no relationship was found between BMI and *ENPP2* methylation in normal samples.

mRNA expression analysis in the same samples showed increased levels in HCC in relation to normal tissues (LogFC: 0.710, FDR: 1.00 × 10^−2^), i.e., the opposite of LC and PC observations, suggesting a methylation-independent and a cancer type-specific regulation of *ENPP2* in HCC. Spearman correlation of mRNA expression (at gene and isoform levels) per CG site revealed the most numerous statistically significant correlations, compared to PC and LC samples, shown in Figure 4C and Table 6. Between gene body CGs, a positive correlation of mRNA expression to cg00320790, cg01243251, cg07236691, cg09444531, cg20048037 and cg20162626 methylation was observed. Apart from the significant correlations established, methylation of the aforementioned CGs was characterized by a linear relationship to *ENPP2* expression, further supporting dependence of the latter on the former. Control samples also showed positive correlation between ENPP2 mRNA expression and methylation of 2 gene body CGs (cg20048037, g20162626). Finally, cg23725583 of body and cg02709432, cg04452959, cg06998282 and cg02156680 of TSS regions showed negative correlation of methylation in relation to ENPP2 mRNA expression. Isoform analysis for the control tissues showed similar correlation patterns (Supplementary Figure S2 and Table S2).

### 3.10. ENPP2 Methylation, Expression and Survival Analysis via UALCAN

**Figure 5:**
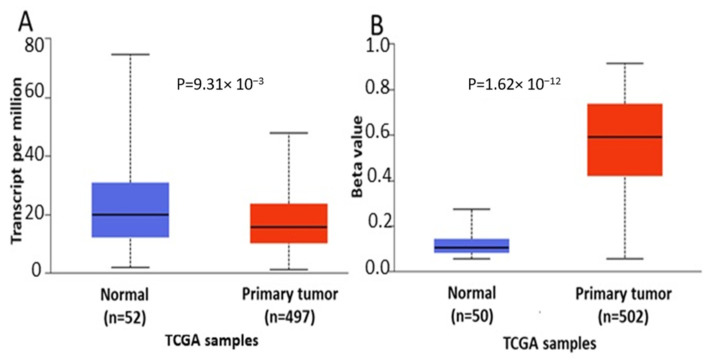
Analysis of *ENPP2* between primary PC tumors and normal tissues using the UALCAN database, concerning (**A**) mRNA expression and (**B**) DNA methylation. Abbreviations: TCGA: The Cancer Genome Atlas, PC: Prostate Cancer.

**Figure 6:**
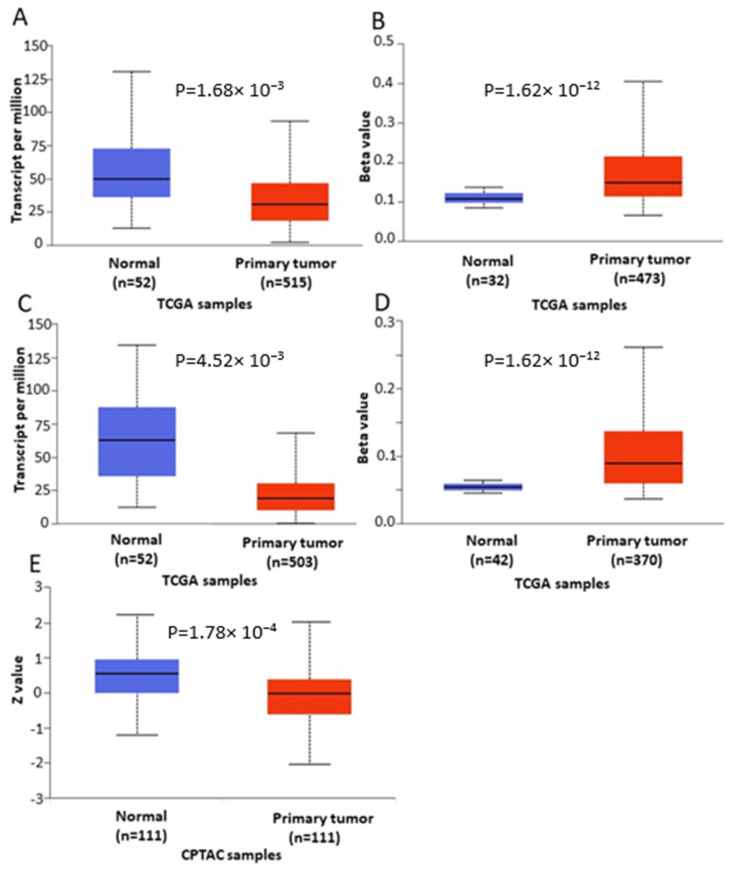
Analysis of *ENPP2* between primary LC tumors and normal tissues using the UALCAN database, concerning (**A**) mRNA expression and (**B**) DNA methylation of adenocarcinoma cases, (**C**) mRNA expression and (**D**) DNA methylation of squamous cell carcinoma and (**E**) protein expression of adenocarcinoma cases. Abbreviations: TCGA: The Cancer Genome Atlas, CPTAC: Clinical Proteomic Tumor Analysis Consortium, LC: Lung Cancer.

**Figure 7:**
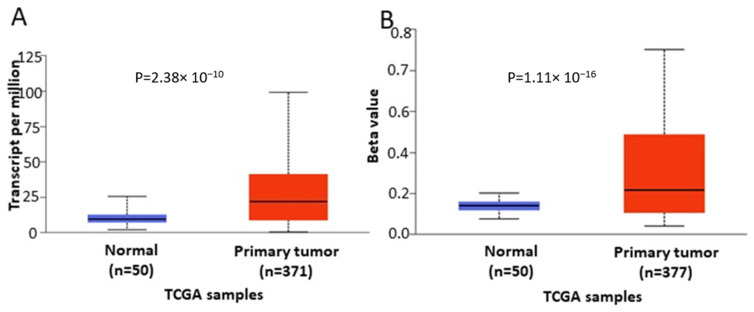
Analysis of *ENPP2* between primary HCC tumors and normal tissues using the UALCAN database, concerning (**A**) mRNA expression and (**B**) DNA methylation. Abbreviations: TCGA: The Cancer Genome Atlas, HCC: Hepatocellular Carcinoma.

In order to further verify our findings, we conducted expression, methylation and survival analysis of *ENPP2* in PC (all adenocarcinoma cases), LC (adenocarcinoma and squamous cell carcinoma cases) and HCC using the UALCAN database. Analysis confirmed the above results as *ENPP2* mRNA was under-expressed in PC (*p* = 9.31 × 10^−3^, Figure 5A) and LC (adenocarcinoma, *p* = 1.68 × 10^−3^ and squamous cell carcinoma, *p* = 4.52 × 10^−3^, Figure 6A,C) and upregulated in HCC (*p* = 2.38 × 10^−10^, Figure 7A). Protein expression analysis was available only for LC adenocarcinoma cases, showing downregulation in primary tumor tissues in relation to normal tissues (Figure 6E, *p* = 1.78 × 10^−4^). Next, methylation analysis revealed upregulation in all cancer types in relation to normal tissues (PC, *p* = 1.62 × 10^−12^, HCC, *p* = 1.11 × 10^−16^ and LC, *p* = 1.62 × 10^−12^ for both types) as depicted in Figure 5, Figure 6 and Figure 7, in accordance with our previous observations. Methylation and expression results via UALCAN strengthen our findings, showing that the *ENPP2* gene is methylated in LC, HCC and PC and this is related to under-expression in LC and PC, suggesting a causative relationship in these two cancer types and a cancer-specific regulatory mechanism in HCC. Finally, survival analysis did not reveal any statistical significance for any of the studied cancers, as depicted in Supplementary Figure S4A–D.

## 4. Discussion

ATX encoded by *ENPP2* is a secreted glycoprotein that forms LPA [42]. The ATX-LPA axis is related to many physiological processes, including embryonic development and wound healing. Dysregulation of ATX expression is connected with various pathological conditions such as cancer, inflammatory diseases and fibrosis [3,4,5]. The exact mechanism by which *ENPP2* expression is regulated is still not fully understood, whereas recently, it has been proved that *ENPP2* is prone to epigenetic alterations [13]. Still, very little information is available about its DNA methylation pattern and the consequent impact in gene expression in health and human pathology.

In the present study we adopted a bioinformatic in silico approach using publicly available datasets from healthy tissues and different cancer tissues and cell lines to analyze methylation patterns of *ENPP2*. Our analysis showed a consistent methylation pattern throughout the gene’s regions across human tissues, i.e., increased methylation in the gene body and decreased methylation in TSS and the 1st exon. Given the fact that *ENPP2* is expressed in almost all tissues and biological fluids [12,43,44], we can postulate that the decreased methylation in the TSS and 1st exon is associated with the active transcription of the gene in most human tissues.

Analysis of cancer datasets revealed aberrant *ENPP2* methylation, showing a malignant-specific profile throughout different cancer types. In general, methylation was increased in the TSS and 1st exon, regions known to hold an important role in gene expression, and decreased in the gene body region. A large number of DMCs were identified between malignant and respective benign tissues. Most importantly, all six DMCs of *ENPP2* located at TSS in the promoter or at the 1st exon showed increased methylation across different cancer types, including HCC, melanoma, CRC, LC and PC. These results corroborate and expand recent observations showing a hypermethylated *ENPP2* promoter in primary tumors of LC and squamous cell carcinoma patients [45] and in breast cancer [13,22,46].

Based on these interesting observations, we next performed in silico analysis of *ENPP2* methylation in datasets retrieved from the TCGA, focusing on those cancer types presenting the greatest number of DMCs, i.e., LC, PC and HCC. TCGA datasets are generally larger compared to those of other research efforts, allowing comparisons of stronger statistical relevance, and most significantly, they contain several clinical and demographic parameters of each patient. In addition, the datasets selected included also mRNA expression data and were therefore suitable for addressing an important objective of this study, i.e., if aberrant methylation is correlated to gene expression. Methylation, clinical and expression data were recovered for the three cancer types. Differential methylation analysis of *ENPP2* revealed that all emerged DMCs identified in transcription-related (TSS and 1st exon) regions were hypermethylated in all three cancers compared to healthy controls, confirming the analysis of the GEO datasets. In addition, the majority of DMCs located at the gene body were hypomethylated in the studied cancers in relation to controls. mRNA levels were decreased in PC and LC in relation to normal tissues. Collectively, our results indicate that the increased methylation of PA and 1st exon CGs is correlated with decreased expression in lung and prostate cancer. This is in line with previous studies in LC and BC showing that *ENPP2* is hypermethylated in tumor tissues in relation to normal, causing down regulation in gene expression [13,45]. In PC, ATX protein was not or was weakly expressed in non-neoplastic epithelial cells and in high-grade intra-epithelial neoplasia, while in cancer cells ATX was only expressed in half of the tumors and was correlated with adverse tumor parameters [47]. A relevant study in LC showed that ATX protein expression and activity was increased in LC tissues and sera [48]. As far as HCC is concerned, our analysis showed upregulation of expression in HCC in relation to normal liver, showing a TSS and 1st exon methylation-independent and a cancer type-specific role of *ENPP2* expression regulation. In a previous study, ATX overexpression in HCC tissues was correlated with inflammation and liver cirrhosis. In addition, liver cancer cell lines presented stronger ATX expression in relation to normal hepatocytes [49]. It should be noted that many authors have demonstrated that the relationship between mRNA expression and protein differs in many cancers. It has been reported in lung cancer and glioblastoma that, for many genes, mRNA expression is lower but protein levels are higher compared with the control [50,51,52,53].

In agreement with the above findings, analysis using the UALCAN database showed that *ENPP2* is hypermethylated and under-expressed in LC and PC, suggesting that DNA methylation regulates expression in LC and PC. However, no regulatory relation was observed between methylation and expression in HCC, as both were upregulated, pointing again to a cancer-specific methylation-independent *ENPP2* regulation. Different mechanisms between cancer types are common. Here, our presented results from the cancer types studied indicate a cancer type-specific profile of *ENPP2* methylation rather than a similar pan-cancer dysregulation. Without availability of suitable methylome datasets or targeted methylation studies of *ENPP2* in each different cancer type, we cannot extrapolate conclusions between cancers.

The same correlation pattern was noticed for *ENPP2* isoforms in all cancer types studied. Interestingly, there was a significant negative correlation between mRNA expression (gene and isoform alpha and beta) and promoter methylation in four CGs (cg02156680, cg02709432, cg04452959 and cg06998282) in PRAD. In LC samples, the methylation of cg06998282 and cg02709432 was negatively correlated with the expression of *ENPP2* and also with isoform beta and gamma. Finally, in the case of HCC, only the methylation of cg06998282 was negatively correlated with the expression of *ENPP2* and isoform beta. The above findings indicate that the promoter methylation of specific CGs is negatively correlated with *ENPP2* and isoform expression differs between cancers, with cg02709432 being a common site in PC and LC but not in the case of HCC. This CG is located at a site that can bind E2F-1 TF, which has been shown to be inhibited by CG methylation [54], and Sp1 TF, which has been found to regulate ENPP2 transcription [55]. Thus, we hypothesized that as the level of methylation increases, methylation of cg02709432 hinders the binding of the TFs to the promoter, thus leading to reduction in ENPP2 gene and isoforms expression.

The expression pattern of isoforms differs between tissues as high expression levels of isoform beta were found in peripheral tissues and plasma, while isoform gamma was mostly found in the brain, and isoform alpha is considered to be the most under-expressed in brain and peripheral tissue in comparison to the other two [56]. According to a relevant study, isoform alpha has a deletion of exon 12, isoform beta a deletion of exons 12 and 21 and isoform gamma a deletion of exon 21 [12], leading to different splice variants. None of the identified DMCs were located at these regions, explaining similar patterns of *ENPP2* mRNA and isoform expression.

DNA methylation within promoters is known to modulate the binding of TFs to regulatory elements, thus resulting in transcriptional repression [57]. In our study, we predicted 39 TFs which can regulate transcription through binding to *ENPP2* promoter’s DMCs. Therefore, any aberrant methylation events in these DMCs during pathological transformation may block TF binding and related transcription. This is further supported by reports involving the identified TFs in *ENPP2* and ATX expression. Indeed, among the predicted TFs, NF kappaB, AP-2 and E2F have been previously shown to be sensitive to CG methylation with consequent inhibition of their DNA binding activities [54]. Another TF predicted to bind DMCs of *ENPP2*, NFAT1, has been shown to mediate ATX overexpression in MDA-MB-435 cells [58]. It has also been shown that blocking the expression of NFAT1 results in downregulation of ATX expression, leading to inhibition of melanoma and metastasis [35]. High C-Jun levels seem to enhance *ENPP2* expression [59]. Interestingly, SP was found to regulate *ENPP2* transcription in neuroblastoma cells by activating a CRE/AP-1-like element at position −142 to −149 and a GAbox at position −227 to −235 near the TSS of *ENPP2* [55]. This is in accordance with our finding that Sp1 can bind near the cg02709432 located at TSS200.

In order to assess any correlation of *ENPP2* methylation to tumor prognosis, clinical characteristics analysis was performed and showed that increased methylation of some CGs was correlated with poor tumor parameters. Indeed, in PC it was associated with larger tumors and non-response to pharmacotherapy, in LC it was connected to the advanced cancer stage and in HCC it was associated with macro-invasion. Hence, *ENPP2* methylation in the identified CGs could be pursued further and be evaluated in clinical cancer samples as biomarkers of cancer progression and poor outcome. In addition, these results corroborate previous data showing that low mRNA expression was associated with worse prognosis in LC [45].

The involvement of *ENPP2* methylation in tumor progression and prognosis was also addressed by analyzing methylomes from cell lines presenting a more or less aggressively invasive phenotype, revealing several DMCs. Higher methylation was observed in the more aggressive in relation to less aggressive HCC and PC cell lines, indicating a connection of *ENPP2* methylation with worse prognostic behavior, in accordance with our findings in the clinical samples.

Finally, analysis of colon cell lines treated with DNA methyltransferase inhibitors showed that 5-AZA caused a decrease of methylation in all CGs in relation to untreated controls in the three studied cell lines, showing a clear demethylation effect in the *ENPP2* gene. Given the contribution of *ENPP2* in a variety of pathologies, further studies could assess a methylation-based reprogramming of *ENPP2* via a variety of methylation inhibitors. Similarly, previous studies have demonstrated that targeting the ATX-LPA-LPP axis is an attractive strategy for introducing new therapeutic choices [60,61].

In conclusion, healthy tissues presented increased methylation of *ENPP2* in the gene body and decreased in the promoter and 1st exon connected to the active transcription of the gene in most human tissues. A different pattern was described in HCC, melanoma, CRC, LC and PC, showing a malignant-specific profile of *ENPP2* methylation. Further analysis of independent TCGA datasets confirmed these results as increased methylation of promoter and 1st exon CGs and decreased *ENPP2* mRNA expression in PC and LC in relation to healthy tissues were found. Furthermore, increased methylation of *ENPP2* was connected to poor prognostic parameters in the same cancers, which was also supported by analysis of cell line datasets. We also found a negative correlation between mRNA expression at gene and isoform levels and methylation of PA CGs that present TF binding sites. In specific, we postulate that the methylation of promoter CGs may hinder the binding of TFs, and thus, the expression of *ENPP2* and isoforms may be reduced.

Our findings contribute to the understanding of methylation events and regulatory mechanism of *ENPP2* in cancer and provide a full description of DMCs to be further validated in functional and clinical studies.

## Figures and Tables

**Table 1:** Methylome datasets retrieved from GEO for the in silico analysis of *ENPP2* methylation.

Dataset	Platform	Compared Patient Groups	References
GSE27130	27 k	236 CRC vs. 236 adjacent colon tissues	[25]
GSE98534	27 k	16 CRC vs. 16 adjacent colon tissues	[26]
GSE63704	450 k	17 LC vs. 43 adjacent lung tissues	-
GSE46306	450 k	6 CC (HPV^+^) vs. 18 CIN3(HPV^+^) vs. 20 normal cervical (HPV^−^) tissues	[27]
GSE134772	EPIC	3 CC(HPV16) vs. 2 CIN1, 1 CIN2, 1 CIN3 (HPV16) vs. 3 normal (HPV^−^) cervical tissues	[28]
GSE120878	450 K	89 melanoma vs. 73 nevus tissues	[29]
GSE76938	450 K	73 PC vs. 63 prostate benign tissues	[30]
GSE97686	450 k	3 GC vs. 3 adjacent gastric vs. 3 normal gastric myofibroblasts	[31]
GSE113017	450 k	30 HCC vs. 30 adjacent liver tissues	[32]
GSE113019	450 k	19 primary HCC vs. 18 recurrent HCC vs. 18 adjacent liver tissues	[32]
GSE71627	450 k	HepG2 vs. SKHep1, LNCaP vs. PC3	[33]
GSE50192	450 k	Adipose abdominal, adipose subcutaneous, splenic artery, bone, bone marrow red, coronary artery, gastric mucosa, lymph node, aorta abdominal, aorta thoracic, bladder, bone marrow yellow, gallbladder, ischiatic nerve, joint cartilage, medulla oblongata, tonsils (basal methylation)	[34,35]
GSE51815	450 k	AZA treated colon cancer cell lines vs. untreated controls	[36]

CRC: Colorectal Cancer; LC: Lung Cancer; CC: Cervical Cancer; HPV^+/−^: Human Papillomavirus positive/negative; CIN: Cervical Intraepithelial Neoplasia; PC: Prostate Cancer; GC: Gastric Cancer; HCC: Hepatocellular Carcinoma.

**Table 2:** DMCs presenting higher or lower methylation in cancer in relation to corresponding control, identified via in silico analysis in *ENPP2*.

Study ID	Compared Groups	CG ID	Mβ Value 1 *	Mβ Value 2 *	Δβ Value ^#^	Regulation	Gene Region	FDR
**HCC**								
GSE113017	Adjacent liver vs. HCC tumor	cg00320790	0.892	0.761	−0.130	Down	Body	2.5 × 10^−2^
		cg07236691	0.832	0.659	−0.173	Down	Body	1.9 × 10^−3^
		cg09444531	0.795	0.619	−0.176	Down	Body	8.6 × 10^−4^
		cg20162626	0.809	0.642	−0.167	Down	Body	3.0 × 10^−2^
		cg02709432	0.123	0.263	0.141	Up	TSS200	1.9 × 10^−2^
		cg04452959	0.058	0.189	0.131	Up	TSS200	2.1 × 10^−2^
		cg02156680	0.067	0.202	0.135	Up	TSS1500	4.0 × 10^−3^
		cg06998282	0.086	0.286	0.200	Up	TSS1500	4.0 × 10^−3^
GSE113019	Adjacent liver vs. primary HCC tumor	cg00320790	0.835	0.623	−0.212	Down	Body	3.0 × 10^−2^
		cg07236691	0.776	0.563	−0.213	Down	Body	4.5 × 10^−2^
		cg09444531	0.721	0.484	−0.237	Down	Body	2.4 × 10^−3^
		cg20048037	0.696	0.474	−0.222	Down	Body	2.1 × 10^−2^
		cg20162626	0.695	0.447	−0.248	Down	Body	7.0 × 10^−3^
		cg23725583	0.718	0.557	−0.161	Down	Body	6.2 × 10^−2^
		cg02156680	0.064	0.188	0.124	Up	TSS1500	1.8 × 10^−2^
		cg06998282	0.105	0.284	0.179	Up	TSS1500	3.0 × 10^−2^
		cg02709432	0.127	0.256	0.129	Up	TSS200	2.0 × 10^−2^
		cg04452959	0.042	0.149	0.106	Up	TSS200	9.3 × 10^−3^
	Adjacent liver vs. (primary & recurrent) HCC tumor	cg00320790	0.835	0.634	−0.201	Down	Body	2.3 × 10^−3^
		cg07236691	0.776	0.568	−0.207	Down	Body	2.3 × 10^−3^
		cg20048037	0.696	0.450	−0.246	Down	Body	1.8 × 10^−3^
		cg23725583	0.718	0.581	−0.137	Down	Body	4.6 × 10^−2^
		cg02156680	0.064	0.198	0.134	Up	TSS1500	4.0 × 10^−3^
		cg06998282	0.105	0.279	0.174	Up	TSS1500	1.3 × 10^−2^
		cg14409958	0.263	0.374	0.111	Up	TSS1500	2.2 × 10^−2^
		cg02709432	0.127	0.253	0.126	Up	TSS200	1.2 × 10^−2^
		cg04452959	0.042	0.141	0.098	Up	TSS200	5.3 × 10^−3^
		cg02534163	0.143	0.274	0.131	Up	1st Exon	2.3 × 10^−3^
**Melanoma**								
GSE120878	Nevus vs. primary melanoma tissues	cg23725583	0.481	0.583	0.102	Up	Body	4.7 × 10^−6^
		cg00320790	0.859	0.834	−0.025	Down	Body	2.3 × 10^−3^
		cg09444531	0.736	0.692	−0.043	Down	Body	1.1 × 10^−2^
		cg20048037	0.801	0.740	−0.061	Down	Body	1.4 × 10^−6^
		cg20162626	0.625	0.575	−0.050	Down	Body	2.3 × 10^−3^
		cg26078665	0.647	0.616	−0.031	Down	Body	8.0 × 10^−3^
		cg04452959	0.105	0.142	0.037	Up	TSS200	5.6 × 10^−4^
		cg02534163	0.155	0.242	0.087	Up	1st Exon	1.4 × 10^−6^
**CRC**								
GSE27130	Adjacent colon vs. CRC	cg14409958	0.201	0.210	0.009	Up	TSS1500	5.6 × 10^−3^
**LC**								
GSE63704	Normal lung vs. LC	cg09444531	0.832	0.774	−0.058	Down	Body	3.0 × 10^−4^
		cg20048037	0.832	0.754	−0.078	Down	Body	5.2 × 10^−5^
		cg20162626	0.863	0.802	−0.061	Down	Body	6.5 × 10^−5^
		cg02709432	0.234	0.265	0.030	Up	TSS200	1.7 × 10^−2^
		cg06998282	0.198	0.268	0.070	Up	TSS1500	2.6 × 10^−3^
		cg14409958	0.264	0.366	0.103	Up	TSS1500	1.4 × 10^−4^
		cg02534163	0.133	0.183	0.050	Up	1st Exon	1.5 × 10^−2^
	IPF vs. LC	cg00320790	0.940	0.904	−0.036	Down	Body	5.6 × 10^−4^
		cg20048037	0.785	0.754	−0.031	Down	Body	5.2 × 10^−1^
		cg20162626	0.844	0.802	−0.042	Down	Body	2.1 × 10^−2^
		cg06998282	0.196	0.268	0.072	Up	TSS1500	4.7 × 10^−3^
		cg14409958	0.269	0.366	0.097	Up	TSS1500	8.4 × 10^−4^
	COPD vs. LC	cg20048037	0.810	0.754	−0.056	Down	Body	8.4 × 10^−3^
		cg20162626	0.850	0.802	−0.048	Down	Body	4.7 × 10^−3^
		cg02709432	0.229	0.265	0.035	Up	TSS200	8.4 × 10^−3^
		cg02156680	0.183	0.204	0.022	Up	TSS1500	3.4 × 10^−2^
		cg06998282	0.184	0.268	0.084	Up	TSS1500	8.4 × 10^−4^
		cg14409958	0.253	0.366	0.114	Up	TSS1500	8.4 × 10^−4^
		cg02534163	0.133	0.183	0.050	Up	1st Exon	2.8 × 10^−2^
**PC**								
GSE76938	benign prostate vs. PC	cg07236691	0.471	0.670	0.199	Up	Body	1.4 × 10^−6^
		cg09444531	0.654	0.775	0.122	Up	Body	1.4 × 10^−6^
		cg23725583	0.845	0.914	0.069	Up	Body	1.0 × 10^−3^
		cg26078665	0.687	0.709	0.022	Up	Body	7.6 × 10^−3^
		cg20162626	0.787	0.687	−0.100	Down	Body	1.4 × 10^−6^
		cg02709432	0.093	0.417	0.324	Up	TSS200	1.4 × 10^−6^
		cg04452959	0.034	0.289	0.255	Up	TSS200	1.4 × 10^−6^
		cg06998282	0.108	0.457	0.349	Up	TSS1500	1.4 × 10^−6^
		cg14409958	0.126	0.399	0.273	Up	TSS1500	1.4 × 10^−6^
		cg02156680	0.070	0.351	0.281	Up	TSS1500	1.4 × 10^−6^
		cg02534163	0.071	0.340	0.268	Up	1st Exon	1.4 × 10^−6^

* Mean β (Mβ) value 1 represents methylation in control and Mean β (Μβ) value 2 methylation in cancer; ^#^ Δβ value: (Mean β value 2-Mean β value 1). Abbreviations: DMC: Differentially Methylated CpG; FDR: False Discovery Rate; HCC: Hepatocellular Carcinoma; TSS: Transcription Start Site; PA: Promoter Associated; CRC: Colorectal Cancer; IPF: Idiopathic Pulmonary Fibrosis; LC: Lung Cancer; COPD: Chronic Obstructive Pulmonary Disease; PC: Prostate Cancer.

**Table 3:** Common DMCs across different cancer types, located at TSS or 1st exon, all presenting upregulation of methylation in relation to respective benign controls.

DMC	Studies Analysed	Cancer Type
cg02156680	GSE113017, GSE113019, GSE63704, GSE76938	HCC, PC
cg02709432	GSE113017, GSE113019, GSE63704, GSE76938	HCC, LC, PC
cg04452959	GSE113017, GSE113019, GSE120878, GSE76938	HCC, melanoma, PC
cg06998282	GSE113017, GSE113019, GSE63704, GSE76938	HCC, LC, PC
cg02534163	GSE113019, GSE120878, GSE63704, GSE76938	HCC, melanoma, LC, PC
cg14409958	GSE113019, GSE27130, GSE63704, GSE76938	HCC, CRC, PC, LC

Abbreviations: CRC: Colorectal Cancer; LC: Lung Cancer; PC: Prostate Cancer; HCC: Hepatocellular Carcinoma.

**Table 4:** DMCs of ENPP2 identified by comparing HCC and PC cell lines with a more (SKHEP1 and PC3 respectively) to less (HEPG2 and LNCAP respectively) invasive phenotype (GSE71627 dataset).

CG ID	Mβ Value 1 *	Mβ Value 2 *	Δβ Value ^#^	Regulation	Gene Region	FDR
**HCC**						
cg00320790	0.808	0.924	0.115	Up	Body	3.1 × 10^−2^
cg09444531	0.353	0.765	0.411	Up	Body	6.5 × 10^−3^
cg20048037	0.564	0.859	0.295	Up	Body	2.4 × 10^−2^
cg07236691	0.689	0.126	−0.563	Down	Body	3.6 × 10^−3^
cg04452959	0.339	0.784	0.444	Up	TSS200	5.1 × 10^−2^
cg02156680	0.472	0.868	0.396	Up	TSS1500	3.2 × 10^−3^
cg06998282	0.637	0.938	0.302	Up	TSS1500	1.8 × 10^−4^
cg02534163	0.710	0.967	0.257	Up	1st Exon	2.7 × 10^−2^
**PC**						
cg00320790	0.574	0.900	0.326	Up	Body	2.3 × 10^−3^
cg07236691	0.778	0.824	0.046	Up	Body	5.6 × 10^−2^
cg09444531	0.251	0.585	0.333	Up	Body	4.3 × 10^−3^
cg20048037	0.370	0.671	0.301	Up	Body	8.8 × 10^−3^
cg20162626	0.190	0.552	0.362	Up	Body	1.7 × 10^−2^
cg26078665	0.662	0.772	0.110	Up	Body	2.3 × 10^−3^
cg02156680	0.725	0.377	−0.348	Down	TSS1500	2.5 × 10^−3^
cg02534163	0.772	0.951	0.179	Up	1st Exon	2.9 × 10^−3^

* Mean β (Mβ) value 1 represents methylation in less invasive cell lines and * Mean β (Μβ) value 2 methylation in more invasive; ^#^ Δβ value: (Mean β value 2-Mean β value 1). Abbreviations: PC: Prostate Cancer, HCC: Hepatocellular Carcinoma, PA: Promoter Associated, TSS: Transcription Start Site.

**Table 5:** Differential methylation and expression analysis of *ENPP2* between normal prostate and PC tumors from TCGA cases.

CG ID	Mβ Value 1 *	Mβ Value 2 *	Δβ Value ^#^	Regulation	Gene Region	FDR
cg07236691	0.568	0.730	0.162	Up	Body	1.44 × 10^−6^
cg09444531	0.697	0.735	0.038	Up	Body	1.09 × 10^−3^
cg20048037	0.885	0.815	−0.070	Down	Body	4.22 × 10^−3^
cg20162626	0.796	0.650	−0.146	Down	Body	1.44 × 10^−6^
cg23725583	0.858	0.877	0.019	Up	Body	9.05 × 10^−3^
cg01243251	0.933	0.904	−0.029	Down	Body	1.44 × 10^−6^
cg14409958	0.251	0.590	0.339	Up	TSS1500	1.44 × 10^−6^
cg02156680	0.156	0.543	0.386	Up	TSS1500	1.44 × 10^−6^
cg06998282	0.212	0.645	0.433	Up	TSS1500	1.44 × 10^−6^
cg02709432	0.213	0.586	0.372	Up	TSS200	1.44 × 10^−6^
cg04452959	0.130	0.466	0.335	Up	TSS200	1.44 × 10^−6^
cg02534163	0.128	0.565	0.436	Up	1st Exon	1.44 × 10^−6^

^#^ Δβ value: (Mean β value 2 * cancer-Mean β value 1 * normal). Abbreviations: PC: Prostate Cancer, FC: Fold Changes.

**Table 6:** Spearman correlation coefficient between *ENPP2* CG methylation and mRNA expression (*p* < 0.05) for PC, LC and HCC samples, showing mainly a negative correlation with PA CG methylation and in most cases a positive correlation with gene body methylation.

Sample Type	CG	Gene Region	Rho	FDR	Correlation	Coefficient *p*-Value
**PC**						
Tumor	cg06998282	TSS1500	−0.253	1.22 × 10^−3^	Negative	1.55 × 10^−2^
	cg02156680	TSS1500	−0.212	2.88 × 10^−3^	Negative	4.43 × 10^−2^
	cg14409958	TSS1500	−0.221	2.18 × 10^−3^	Negative	3.90 × 10^−2^
	cg02709432	TSS200	−0.176	1.30 × 10^−2^	Negative	4.39 × 10^−2^
	cg02534163	1st Exon	−0.226	2.18 × 10^−3^	Negative	1.30 × 10^−2^
**LC**						
Tumor	cg06998282	TSS1500	−0.142	>0.05	Negative	3.74 × 10^−3^
	cg14409958	TSS1500	−0.213	2.19 × 10^−2^	Negative	1.05 × 10^−4^
Control	cg07236691	Body	−0.564	>0.05	Negative	2.31 × 10^−2^
**HCC**						
Tumor	cg00320790	Body	0.297	9.46 × 10^−6^	Positive	6.29 × 10^−5^
	cg01243251	Body	0.247	2.94 × 10^−4^	Positive	6.90 × 10^−4^
	cg07236691	Body	0.239	4.06 × 10^−4^	Positive	2.28 × 10^−4^
	cg09444531	Body	0.395	1.22 × 10^−9^	Positive	1.53 × 10^−7^
	cg20048037	Body	0.436	8.81 × 10^−12^	Positive	1.02 × 10^−8^
	cg20162626	Body	0.473	0.00× 10^0^	Positive	2.10 × 10^−9^
	cg06998282	TSS1500	−0.137	>0.05	Negative	4.55 × 10^−2^
Control	cg20162626	Body	0.422	2.63 × 10^−2^	Positive	3.39 × 10^−2^
	cg23725583	Body	−0.35	4.74 × 10^−2^	Negative	2.61 × 10^−2^
	cg02709432	TSS200	−0.361	4.56 × 10^−2^	Negative	3.25 × 10^−2^
	cg04452959	TSS200	−0.411	2.63 × 10^−2^	Negative	2.87 × 10^−2^
	cg06998282	TSS1500	−0.464	1.60 × 10^−2^	Negative	4.53 × 10^−3^
	cg02156680	TSS1500	−0.393	2.99 × 10^−2^	Negative	3.52 × 10^−2^

**Table 7:** Differential methylation and expression analysis of *ENPP2* between normal lung and LC tumors from TCGA cases.

CG ID	Mβ Value 1 *	Mβ Value 2 *	Δβ Value ^#^	Regulation	Gene Region	FDR
cg20162626	0.750	0.636	−0.114	Down	Body	1.19 × 10^−2^
cg20048037	0.722	0.624	−0.098	Down	Body	5.18 × 10^−2^
cg07236691	0.561	0.547	−0.014	Down	Body	2.95 × 10^−4^
cg09444531	0.730	0.630	−0.100	Down	Body	2.95 × 10^−4^
cg04452959	0.071	0.138	0.066	Up	TSS200	7.19 × 10^−3^
cg14409958	0.105	0.262	0.158	Up	TSS1500	2.95 × 10^−4^
cg06998282	0.096	0.230	0.134	Up	TSS1500	1.05 × 10^−2^
cg02534163	0.109	0.255	0.145	Up	1st Exon	8.19 × 10^−4^

Mβ Value 1 *: Mean β value normal, Mβ Value 2 *: Mean β value cancer ^#^ Δ β value: (Mean β value 2 * cancer-Mean β value 1 * normal). Abbreviations: LC: Lung Cancer, FC: Fold Changes.

**Table 8:** Differential methylation and expression analysis of *ENPP2* between normal liver and HCC tumors from TCGA cases.

CG ID	Mβ Value 1 *	Mβ Value 2 *	Δβ Value ^#^	Regulation	Gene Region	FDR
cg00320790	0.929	0.798	−0.131	Down	Body	3.90 × 10^−5^
cg01243251	0.917	0.872	−0.046	Down	Body	1.99 × 10^−3^
cg07236691	0.878	0.734	−0.143	Down	Body	1.30 × 10^−5^
cg09444531	0.850	0.689	−0.161	Down	Body	1.30 × 10^−5^
cg20048037	0.832	0.684	−0.148	Down	Body	4.04 × 10^−2^
cg20162626	0.833	0.671	−0.161	Down	Body	3.75 × 10^−4^
cg23725583	0.864	0.765	−0.099	Down	Body	1.50 × 10^−2^
cg04452959	0.054	0.180	0.126	Up	TSS200	4.46 × 10^−2^
cg02709432	0.104	0.264	0.160	Up	TSS200	2.83 × 10^−4^
cg02156680	0.065	0.210	0.144	Up	TSS1500	3.76 × 10^−2^
cg06998282	0.116	0.300	0.184	Up	TSS1500	1.86 × 10^−2^
cg14409958	0.340	0.463	0.123	Up	TSS1500	1.86 × 10^−2^
cg02534163	0.070	0.247	0.177	Up	1st Exon	3.15 × 10^−4^

Mβ Value 1 *: Mean β value normal, Mβ Value 2 *: Mean β value cancer ^#^ Δβ value: (Mean β value cancer-Mean β value normal). Abbreviations: LC: Lung Cancer, FC: Fold Changes.

## Data Availability

Data are available upon request.

## Supplementary Materials

The following are available online at https://www.mdpi.com/article/10.3390/ijms222111958/s1.
