# Supplementary material for: ENPP2 Methylation in Health and Cancer
Source: Int J Mol Sci. 2021 Nov 4;22(21):11958. doi: 10.3390/ijms222111958 (PMC8585013; doi:10.3390/ijms222111958)
Supplement: Supplementary file 1 [file ijms-22-11958-s001.zip › suppl. files/SUPPL. TABLE 1.pdf]

Supplementary Table 1: DMCs identified in *ENPP2* when comparing methylomes of CRC cell lines before and after treatment with 5-Aza-CdR (GSE51815 dataset). All studied CG sites were a demethylated as shown by the negative Δβ values.

CG ID	Mβ value 1*	Mβ value 2*	Δβ value#	Location relative to gene	FDR
**HT116 1KO vs AZA treated**					
cg00320790	0.785	0.595	-0.190	Body	1.9E-02
cg07236691	0.799	0.592	-0.207	Body	2.5E-03
cg09444531	0.391	0.288	-0.103	Body	6.1E-03
cg20048037	0.510	0.365	-0.146	Body	3.8E-02
cg20162626	0.543	0.412	-0.131	Body	2.5E-03
cg23725583	0.578	0.424	-0.154	Body	7.7E-05
cg26078665	0.825	0.749	-0.076	Body	8.5E-03
cg04452959	0.171	0.150	-0.021	TSS200	8.0E-03
cg06998282	0.708	0.458	-0.251	TSS1500	2.5E-03
cg14409958	0.463	0.332	-0.131	TSS1500	1.9E-02
cg02534163	0.468	0.181	-0.287	1st Exon	7.7E-05
**HT116 3BKO vs AZA treated**					
cg00320790	0.946	0.678	-0.268	Body	1.2E-03
cg01243251	0.930	0.812	-0.118	Body	3.2E-02
cg07236691	0.835	0.515	-0.319	Body	2.8E-04
cg09444531	0.617	0.403	-0.214	Body	2.0E-02
cg20048037	0.842	0.518	-0.325	Body	2.9E-04
cg20162626	0.752	0.463	-0.288	Body	2.0E-02
cg23725583	0.928	0.657	-0.270	Body	1.5E-04
cg26078665	0.873	0.752	-0.122	Body	7.3E-04
cg02709432	0.417	0.263	-0.154	TSS200	4.4E-04
cg04452959	0.581	0.347	-0.234	TSS200	1.1E-04
cg02156680	0.188	0.106	-0.082	TSS1500	6.3E-04
cg06998282	0.953	0.571	-0.381	TSS1500	2.0E-04
cg14409958	0.768	0.395	-0.373	TSS1500	2.2E-04
cg02534163	0.958	0.584	-0.374	1st Exon	2.2E-04
**HT116 WT vs AZA treated**					
cg00320790	0.873	0.668	-0.206	Body	5.6E-03

cg01243251	0.894	0.769	-0.125	Body	1.0E-04
cg07236691	0.815	0.610	-0.205	Body	9.6E-03
cg09444531	0.860	0.598	-0.262	Body	4.7E-03
cg20048037	0.654	0.543	-0.110	Body	9.6E-03
cg20162626	0.613	0.483	-0.130	Body	1.4E-02
cg23725583	0.774	0.615	-0.160	Body	3.2E-02
cg26078665	0.856	0.740	-0.116	Body	1.4E-02
cg02709432	0.447	0.361	-0.087	TSS200	3.4E-02
cg04452959	0.526	0.389	-0.138	TSS200	5.6E-03
cg06998282	0.887	0.618	-0.269	TSS1500	1.0E-03
cg14409958	0.705	0.474	-0.231	TSS1500	1.3E-02

*Mean β (Mβ) value 1 represents methylation without 5-Aza-CdR treatment and *Mean β (Mβ) value 2 methylation in 5-Aza-CdR treated cell lines; #Δ β value: (Mean β value 2-Mean β value 1)

Abbreviations: PA: Promoter Associated, TSS: Transcription Start Site, 5-Aza-CdR: 5-AZA-2'-deoxycytidine, HT116 1KO: colon cancer cell line, knockout of the DNA methyltransferase gene *DNMT1*, HT116 3BKO: colon cancer cell line, knockout of the DNA methyltransferase gene *DNMT3B*
