# Supplementary material for: ENPP2 Methylation in Health and Cancer
Source: Int J Mol Sci. 2021 Nov 4;22(21):11958. doi: 10.3390/ijms222111958 (PMC8585013; doi:10.3390/ijms222111958)
Supplement: Supplementary file 1 [file ijms-22-11958-s001.zip › suppl. files/SUPPL. TABLE 2.pdf]

Supplementary Table 2: Spearman correlation coefficient and the kind of correlation between methylation of CGs and each isoform expression of PC, LC and HCC (control and tumor samples). TSS and 1st Exon CGs presented negative correlations, while positive correlations emerged for the gene body CGs.

Sample type	Isoform	CpG	Location	Rho	p-value	Correlation
			**PC**			
Tumor	uc003yos.1	cg07236691	Body	-0,203	3,16E-03	Negative
		cg02709432	TSS200	-0,163	1,77E-02	Negative
		cg04452959	TSS200	-0,195	4,50E-03	Negative
		cg02156680	TSS1500	-0,202	3,26E-03	Negative
		cg06998282	TSS1500	-0,237	5,67E-04	Negative
		cg14409958	TSS1500	-0,192	5,11E-03	Negative
		cg02534163	1stExon	-0,227	9,44E-04	Negative
Control		cg00320790	Body	0,36	3,71E-02	Negative
		cg04452959	TSS200	-0,439	9,98E-03	Negative
		cg02534163	1stExon	-0,359	3,78E-02	Negative
Tumor	uc003yot.1	cg07236691	Body	-0,202	0,002022	Negative
		cg02709432	TSS200	-0,167	0,011268	Negative
		cg04452959	TSS200	-0,17	0,009807	Negative
		cg02156680	TSS1500	-0,185	0,004898	Negative
		cg06998282	TSS1500	-0,216	0,000982	Negative
		cg14409958	TSS1500	-0,197	0,002625	Negative
		cg02534163	1stExon	-0,206	0,001639	Negative
Control		cg04452959	TSS200	-0,436	0,009462	Negative
		cg02156680	TSS1500	-0,338	0,047755	Negative
Tumor	uc003yor.1	cg07236691	Body	-0,232	3,78E-04	Negative
		cg02709432	TSS200	-0,152	2,06E-02	Negative
		cg04452959	TSS200	-0,153	1,95E-02	Negative
		cg02156680	TSS1500	-0,191	3,50E-03	Negative
		cg06998282	TSS1500	-0,241	2,15E-04	Negative
		cg14409958	TSS1500	-0,212	1,16E-03	Negative
		cg02534163	1stExon	-0,209	1,37E-03	Negative
Control		cg07236691	Body	0,371	2,87E-02	Positive
Tumor	uc010mdd.1	cg23725583	Body	0,215	0,032688	Positive
		cg04452959	TSS200	0,042	0,680244	Negative
			**LC**			
Tumor	uc003yos.1	cg14409958	TSS1500	-0,144	0,034478	Negative
Control		cg20048037	Body	0,539	0,040659	Positive
Tumor	uc003yot.1	cg23725583	Body	-0,163	0,016612	Negative
		cg14409958	TSS1500	-0,216	0,001482	Negative
		cg06998282	TSS1500	-0,152	0,02604	Negative
		cg02534163	1stExon	-0,16	0,018625	Negative
Control		cg23725583	Body	-0,618	0,01631	Negative
		cg07236691	Body	-0,539	0,040659	Negative
		cg02709432	TSS200	-0,55	0,036284	Negative
Tumor	uc003yor.1	cg07236691	Body	0,154	0,02429	Positive
		cg14409958	TSS1500	-0,156	0,022303	Negative

Tumor	uc010mdd.1	cg14409958	TSS1500	-0,152	0,025406	Negative
Control		cg23725583	Body	-0,529	0,042578	Negative
		cg09444531	Body	0,542	0,037063	Positive
		cg02709432	TSS200	-0,543	0,036321	Negative
**HCC**						
Tumor	uc003yos.1	cg20162626	Body	0,241	1,60E-04	Positive
		cg09444531	Body	0,159	1,34E-02	Positive
		cg20048037	Body	0,158	1,41E-02	Positive
Control		cg20162626	Body	0,323	3,93E-02	Positive
		cg06998282	TSS1500	-0,555	1,64E-04	Negative
		cg14409958	TSS1500	-0,389	1,19E-02	Negative
		cg02156680	TSS1500	-0,381	1,41E-02	Negative
Tumor	uc003yot.1	cg20162626	Body	0,402	1,00E-08	Positive
		cg09444531	Body	0,33	1,56E-07	Positive
		cg20048037	Body	0,327	2,14E-07	Positive
		cg00320790	Body	0,259	4,63E-05	Positive
		cg01243251	Body	0,215	8,01E-04	Positive
		cg07236691	Body	0,161	1,21E-02	Positive
		cg14409958	TSS1500	-0,15	2,02E-02	Negative
		cg06998282	TSS1500	-0,137	3,34E-02	Negative
Control		cg20162626	Body	0,399	1,02E-02	Positive
		cg20048037	Body	0,349	2,60E-02	Positive
		cg02709432	TSS200	-0,332	3,44E-02	Negative
		cg04452959	TSS200	-0,363	2,01E-02	Negative
		cg06998282	TSS1500	-0,478	1,78E-03	Negative
		cg02156680	TSS1500	-0,397	1,07E-02	Negative
Tumor	uc003yor.1	cg20162626	Body	0,468	1,00E-08	Positive
		cg20048037	Body	0,393	2,61E-10	Positive
		cg09444531	Body	0,379	1,24E-09	Positive
		cg00320790	Body	0,287	5,79E-06	Positive
		cg01243251	Body	0,252	7,77E-05	Positive
		cg07236691	Body	0,195	0,002347	Positive
		cg06998282	TSS1500	-0,131	0,042044	Negative
Control		cg20162626	Body	0,379	0,014982	Positive
		cg23725583	Body	-0,366	0,019074	Negative
		cg20048037	Body	0,356	0,023067	Positive
		cg04452959	TSS200	-0,426	0,0058	Negative
		cg02709432	TSS200	-0,371	0,017616	Negative
		cg06998282	TSS1500	-0,358	0,022025	Negative
		cg02156680	TSS1500	-0,356	0,022785	Negative
Tumor	uc010mdd.1	cg20162626	Body	0,253	6,91E-05	Positive
		cg09444531	Body	0,239	0,000183	Positive
		cg20048037	Body	0,186	0,003827	Positive
		cg00320790	Body	0,162	0,011713	Positive

Abbreviations: TSS: Transcription Start Site
