# Supplementary figures and images for: ENPP2 Methylation in Health and Cancer
Source: Int J Mol Sci. 2021 Nov 4;22(21):11958. doi: 10.3390/ijms222111958 (PMC8585013; doi:10.3390/ijms222111958)

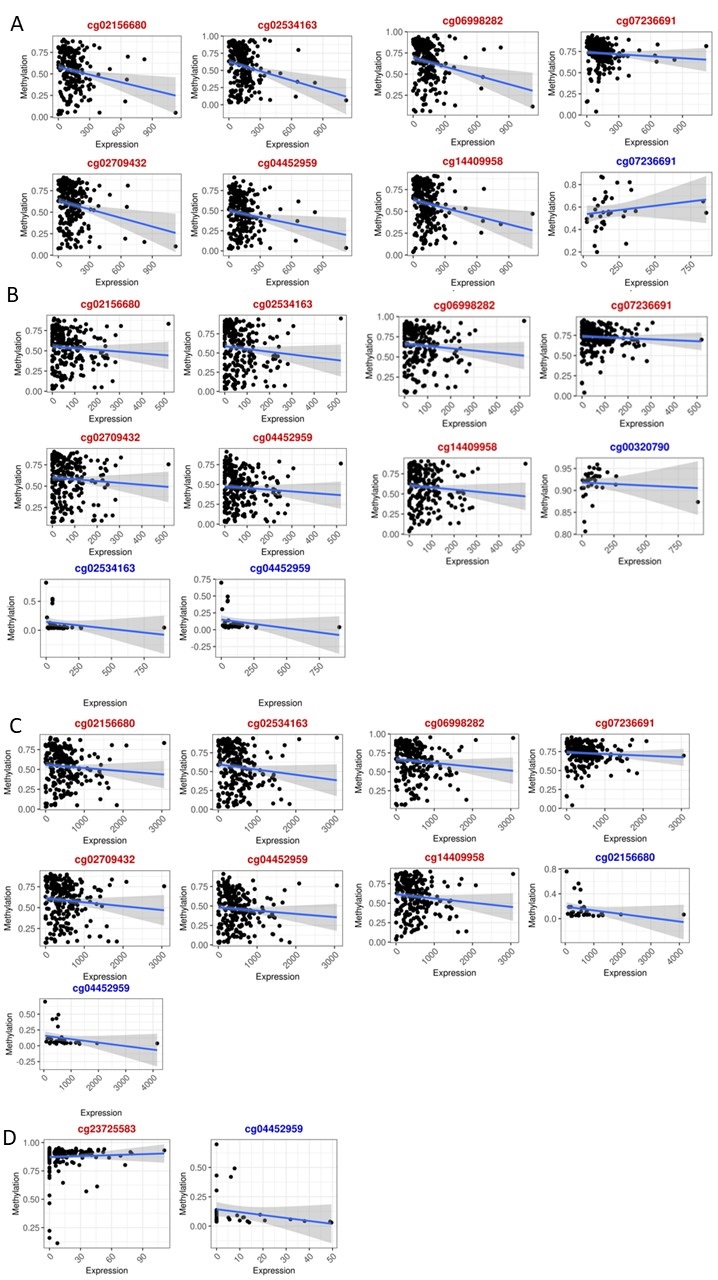

Supplement: Supplementary file 1 [file ijms-22-11958-s001.zip › suppl. files/SUPPL. FIGURE 1.jpg]

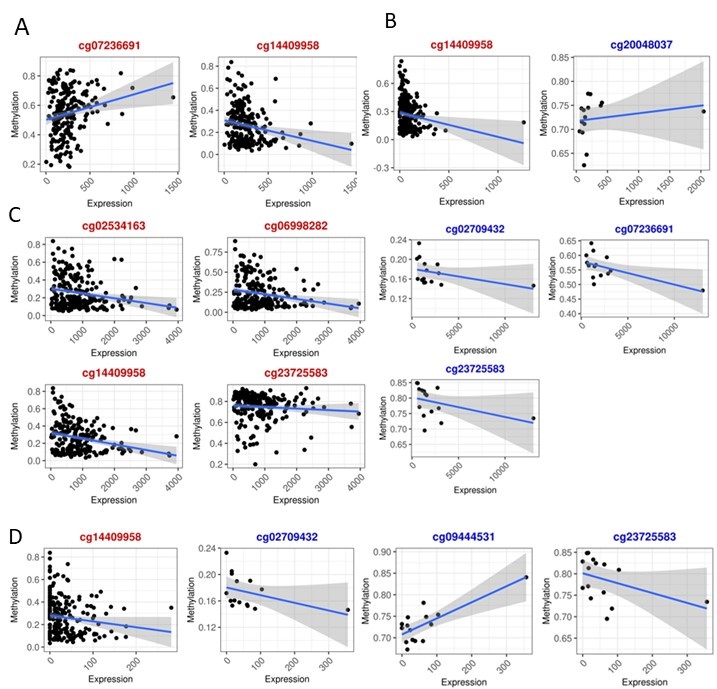

Supplement: Supplementary file 1 [file ijms-22-11958-s001.zip › suppl. files/SUPPL. FIGURE 2.jpg]

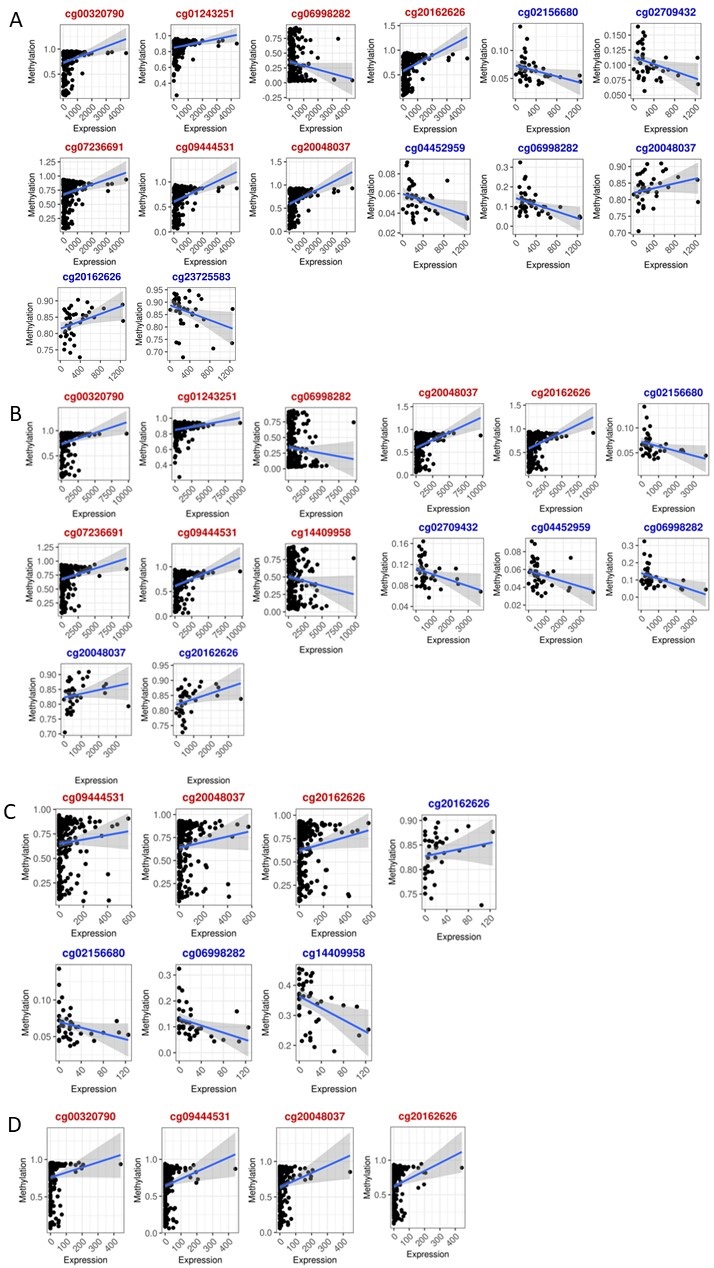

Supplement: Supplementary file 1 [file ijms-22-11958-s001.zip › suppl. files/SUPPL. FIGURE 3.jpg]

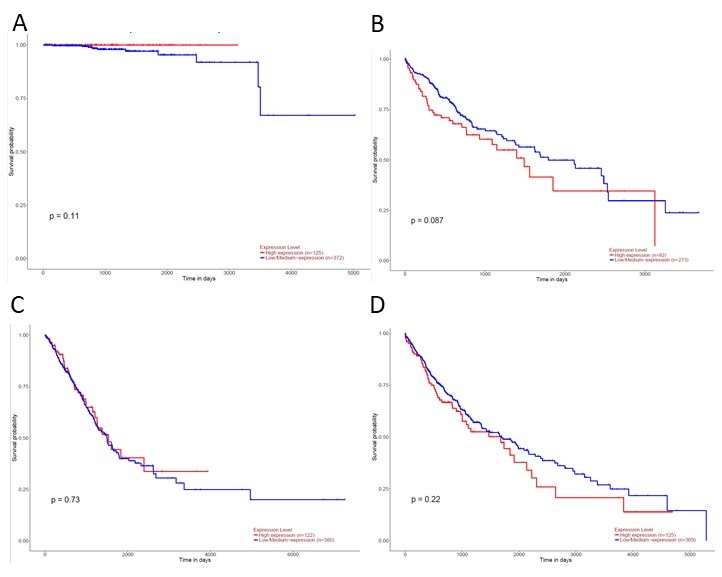

Supplement: Supplementary file 1 [file ijms-22-11958-s001.zip › suppl. files/SUPPL. FIGURE 4.jpg]
